# Understanding the Principles of Pattern Formation Driven by Notch Signaling by Integrating Experiments and Theoretical Models

**DOI:** 10.3389/fphys.2020.00929

**Published:** 2020-07-31

**Authors:** Federico Bocci, José Nelson Onuchic, Mohit Kumar Jolly

**Affiliations:** ^1^Center for Theoretical Biological Physics, Rice University, Houston, TX, United States; ^2^Department of Physics and Astronomy, Rice University, Houston, TX, United States; ^3^Department of Chemistry, Rice University, Houston, TX, United States; ^4^Department of Biosciences, Rice University, Houston, TX, United States; ^5^Centre for BioSystems Science and Engineering, Indian Institute of Science, Bengaluru, India

**Keywords:** Notch, Delta, Jagged, lateral inhibition, lateral induction, spatial pattern, mathematical modeling, cell-cell signaling

## Abstract

Notch signaling is an evolutionary conserved cell-cell communication pathway. Besides regulating cell-fate decisions at an individual cell level, Notch signaling coordinates the emergent spatiotemporal patterning in a tissue through ligand-receptor interactions among transmembrane molecules of neighboring cells, as seen in embryonic development, angiogenesis, or wound healing. Due to its ubiquitous nature, Notch signaling is also implicated in several aspects of cancer progression, including tumor angiogenesis, stemness of cancer cells and cellular invasion. Here, we review experimental and computational models that help understand the operating principles of cell patterning driven by Notch signaling. First, we discuss the basic mechanisms of spatial patterning via canonical lateral inhibition and lateral induction mechanisms, including examples from angiogenesis, inner ear development and cancer metastasis. Next, we analyze additional layers of complexity in the Notch pathway, including the effect of varying cell sizes and shapes, ligand-receptor binding within the same cell, variable binding affinity of different ligand/receptor subtypes, and filopodia. Finally, we discuss some recent evidence of mechanosensitivity in the Notch pathway in driving collective epithelial cell migration and cardiovascular morphogenesis.

## Introduction

**FIGURE 1:**
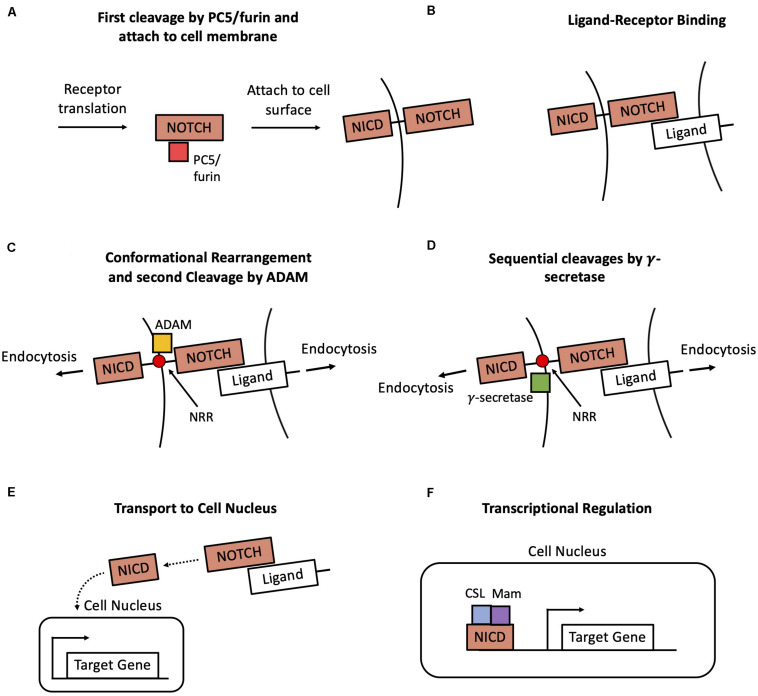
Overview of the Notch transduction pathway. **(A)** A newly produced Notch molecule undergoes a first cleavage by PC5/furin and then attaches to the cell membrane as a transmembrane receptor. **(B)** The Notch transmembrane receptor binds to a ligand at the surface of a neighbor cell. **(C)** Pulling forces originated in both cells expose the Negative Regulatory Region (NRR) of the receptor, hence enabling a cleavage by ADAM. **(D)** Afterward, the receptor undergoes two successive cleavages by γ-secretase, thus leading to the release the Notch Intracellular Domain (NICD) in the cytoplasm. **(E)** NICD is transported to the cell nucleus. **(F)** NICD transcriptionally regulates several target genes in cooperation with other co-activators such as CSL and Mastermind (Mam).

Notch signaling is central in cell fate decisions and therefore it is one of the most well-conserved transduction pathways in metazoans (Bray, 2016). In its simpler form, the signaling cascade includes only a limited number of well-conserved steps, including ligand binding to the Notch transmembrane receptor, release of the Notch intracellular domain (NICD) in the cytoplasm, and downstream regulation of NICD on its target genes (Figure 1; Bray, 2016; Kovall et al., 2017; Sjöqvist and Andersson, 2019). Despite this simplicity, Notch regulates a multitude of different biological processes including cell differentiation, proliferation and death (Bray, 2016).

Each of the abovementioned steps in this cascade raises unanswered questions that would improve our understanding of several developmental processes and may also provide key insights to alleviate many pathological conditions, including cancer (Li et al., 2014; Bray, 2016; Kovall et al., 2017; Siebel and Lendahl, 2017; Sjöqvist and Andersson, 2019). Here, we explicitly focus on the role of Notch signaling in coordinating cell fate decisions and patterning at a multicellular level, and how various experimental and computational models can be integrated to elucidate the underlying dynamical principles of pattern formation. Due to its multi-cellular nature, Notch signaling offers an opportunity to understand how cell-fate decision in individual cells may be relayed to generate emergent multi-cellular dynamics. Different Notch ligands can orchestrate different principles of multicellular spatial patterning via different positive and negative feedback regulation between NICD and its transcriptional targets (Bray, 2006). For instance, Notch signaling can coordinate a divergent cell fate between two neighboring cells, a process known as lateral inhibition (Bray, 2016). Moreover, Notch can modulate the opposite process, the lateral induction (Hartman et al., 2010; Petrovic et al., 2014), by coordinating a similar cell state among neighbors.

In this review, we offer a bird’s eye view on how to interpret cell-level and tissue-level dynamics with simple concepts such as lateral inhibition and lateral induction, discuss the limitations of these models, and highlight a novel set of questions that require integrating experimental investigation with concepts from quantitative mechanistic modeling. In doing so, we bring together the analysis of several biological systems as well as theoretical modeling approaches that highlight the emergence of common themes in the Notch pathway. For the sake of simplicity, technical details of the underlying biology and mathematical models have been occasionally omitted, and relevant literature has been suggested. Furthermore, given the extensive set of topics covered in this review, we have focused on certain experimental and/or theoretical models that are representative of a particular system, and pointed the interested readers to relevant reviews for in-depth discussions of specific areas of research.

First, we review some aspects of the Notch signaling cascade that are necessary to understand Notch-driven pattern formation. It is followed by a discussion of various modeling approaches that can be used to understand the operating principles of Notch. After these two introductory sections, we discuss the principles of Notch-driven patterning. We analyze how Notch signaling gives rise to divergent cell fate – lateral inhibition – and convergent cell fate – lateral induction – among neighboring cells. Experimental evidence and theoretical modeling have contributed to understanding the competition and synergy between these patterning mechanisms in various physiological and pathological systems, including angiogenesis, inner ear development and cancer metastasis. Moreover, we review the oscillatory dynamics of Notch signaling that can arise due to coupling with other signaling pathways, for instance, during somitogenesis. Further, we examine the role of various molecular and morphological features that introduce additional layers of complexity to the canonical Notch signaling outcomes. The scenarios discussed here include the role of cell shape and packing geometry, cis-interactions between molecules within the same cell, mechanisms that alter the binding affinity between ligand and receptor paralogs, and beyond-nearest neighbor signaling through filopodia. In the final section, we review evidence pointing to a role for mechanosensitivity in assisting Notch-driven cell-fate decision. Relevant examples discussed here include collective epithelial cell migration and cardiovascular morphogenesis.

## Overview of Notch Signaling

In this section, we discuss the main components and steps of the Notch signaling cascade. We will avoid excessive details on the molecular structure of the Notch receptor and ligands that are not required for the topics discussed in this review.

The main steps of the Notch signaling cascade are very well conserved across several organisms and include production and targeting of the Notch receptor to the cell membrane, ligand-receptor binding, conformational rearrangement of the receptor, release of the intracellular domain (NICD) and downstream transcriptional regulation (Figure 1). First, a newly produced Notch receptor molecule is glycosylated by the enzymes O-fut and Rumi, and successively subjected to proteolytic cleavage by the PC5/furin at site 1 (S1) (Kopan and Ilagan, 2009). Afterward, the mature Notch molecule attaches to the cell surface as a transmembrane receptor (Figure 1A). The signaling is initiated with the binding of an extracellular ligand to the transmembrane Notch receptor (Figure 1B). Typically, the ligand is a transmembrane protein at the surface of a neighboring cell (juxtacrine signaling), but it can occasionally be a soluble ligand in the extracellular microenvironment (paracrine signaling) (D’Souza et al., 2010). In particular, two classes of ligands, referred to as Delta-like and Jagged-like, can bind to the Notch receptors. The ligand-receptor binding and forces originated by endocytosis induce a conformational change in the structure of the Notch receptor. This modification exposes a previously shielded region of the receptor, the Negative regulatory region (NRR). Following this conformational change, the receptor sequentially undergoes a cleavage by the enzymes ADAM at site 2 (S2, Figure 1C) and two cleavages by γ-secretase at sites 3 and 4 (S3–S4, Figure 1D; Kopan and Ilagan, 2009), resulting in the release of the NICD in the cytoplasm (Figures 1B,C). The NICD translocates to the cell nucleus, where it regulates several target genes together with cooperating transcriptional cofactors such as CSL and Mastermind (Mam) (Figures 1E,F; Bray, 2016).

Notably, NICD regulates the transcription of the Notch receptor and its ligands, either in a direct or indirect manner. Specifically, NICD promotes the transcription of Hey/Hes1 (Shimojo et al., 2011) – an inhibitor of Delta – while directly activating Notch and Jagged (Manderfield et al., 2012). Therefore, Notch signaling introduces a biochemical feedback between neighboring cells that coordinates their cell fate decision (Shaya and Sprinzak, 2011; Sjöqvist and Andersson, 2019). The implications of these biochemical feedbacks in multicellular patterning are the subject of the section about “Spatiotemporal Patterning Guided by Notch Signaling.”

Although the main steps of the signaling are quite general, there are specific aspects that differ from one organism to another, or even from cell to cell – these will be the focus of the section on “Non-canonical Modulation of Notch Signaling.” First, the signaling depends on cell-cell contact area because physical contact is required for juxtacrine signaling (Shaya et al., 2017). We will discuss how cell size modulates Notch signaling and plays a role in determining cell fate. Furthermore, ligands and receptors can bind within the same cell, thus leading to degradation of the ligand-receptor complex without release of NICD (referred as cis-inhibition). Despite not leading to NICD release, cis-inhibition plays a pivotal role by sequestering ligands and receptors that would otherwise contribute to active signaling (Celis and de Bray, 1997; Sprinzak et al., 2010). Third, different model organisms have different number of ligand and receptor subtypes. Drosophila melanogaster –where Notch was firstly extensively characterized – has one type of Notch receptor and two types of ligands (Delta and Serrate, equivalent of Jagged). Conversely, most mammalian organisms have four Notch paralogs (Notch1, Notch2, Notch3, Notch4), three Delta-like ligands (Dll1, Dll3, Dll4) and two Jagged-like ligands (Jagged1, Jagged2). Table 1 offers a comparison of the main components of the signaling between several popular model organisms. Different pairs of ligand and receptor subtypes possess different binding affinities and have been even associated with different biological functions (Bray, 2016; Sjöqvist and Andersson, 2019). Finally, the signaling can be occasionally extended beyond nearest neighbors via filopodia that introduce transient contacts between second or third-nearest neighbor cells, as see for instance in hair cell patterning during Drosophila wing development (Cohen et al., 2010).

**TABLE 1:** List of Notch molecular components, examples of biological processes regulated by Notch signaling, and references to examine in depth Notch signaling for various organisms.

**Organism**	**Main components**	**Example of biological process**	**References**
*Drosophila Melanogaster* (fruitfly)	Notch Delta Serrate	Wing disk formation, bristle patterning	Bray, 2016; Sjöqvist and Andersson, 2019
*Caenorhabditis elegans* (roundworm)	Lin-12, glp-1 Apx-1 Lag-2	Vulval precursor cell specification	Greenwald, 1998
*Danio rerio* (Zebrafish)	Notch 1, 2 Delta A, B, C, D Jagged 1, 2	Somitogenesis, artery and vein specification	Lawson et al., 2001; Venzin and Oates, 2019
*Gallus gallus domesticus* (chicken)	Notch 1, 2 Delta-like 1, 4 Jagged/Serrate 1, 2	Inner ear development	Neves et al., 2013
*Mus musculus* (house mouse)	Notch 1, 2, 3, 4 Delta 1, 3, 4 Jagged 1, 2	Inner ear development, vascular smooth muscle cell development	Bray, 2016; Sjöqvist and Andersson, 2019
*Homo sapiens*	Notch 1, 2, 3, 4 Delta 1, 3, 4 Jagged 1, 2	Inner ear development, vascular smooth muscle cell development	Bray, 2016; Sjöqvist and Andersson, 2019

Further details on the signaling cascade will not be considered here; additional information can be found in several excellent reviews (Kopan and Ilagan, 2009; Bray, 2016; Kovall et al., 2017; Sjöqvist and Andersson, 2019). Here, we focus on generic principles of multicellular patterning obtained via Notch signaling as a whole.

## Mathematical Formalism to Describe Notch Signaling

In this section, we briefly overview different classes of theoretical models that have been applied to Notch signaling.

Many mathematical models aim at reconstructing the dynamics of mutually interacting biochemical species and/or genes in the Notch pathway with ordinary differential equations (ODEs). In these models, each chemical species/gene is described by a variable (*X*), which can either represent a concentration or copy number. In many cases, since molecular copy numbers are large, *X* is treated as a continuous variable that obeys an ODE of the form:

(1) $$\frac{d\,X}{d\,t}=K_{\text{prod}}-\Gamma_{\text{Degr}}$$

In this equation, *K*_*prod*_ represents any biochemical process that regulates the production of *X*, potentially including constitutive transcription, transcriptional activation or inhibition, translation and any other post-translational interaction that might be relevant in a specific system. Transcriptional regulation of NICD on the Notch receptors and ligands is typically described with Hill functions:

(2) $$K_{\text{prod}}=K_{0}\,\frac{1+\lambda \,{\left(\frac{\text{NICD}}{S_{0}}\right)}^{n}}{1+{\left(\frac{\text{NICD}}{S_{0}}\right)}^{n}}\;$$

**FIGURE 2:**
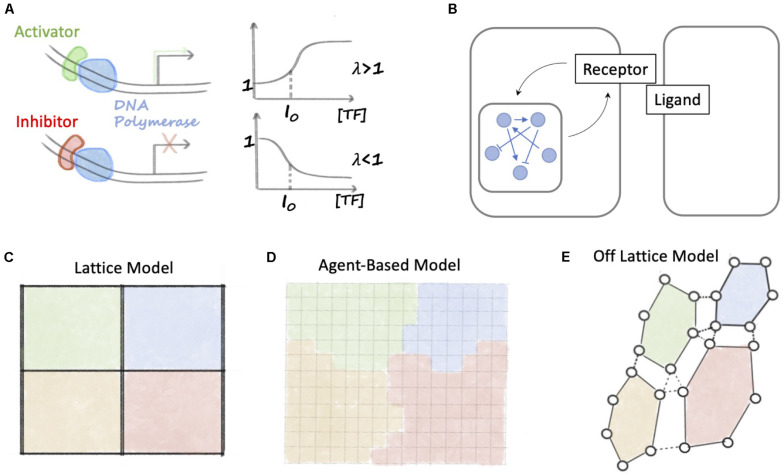
Overview of mathematical methods to study Notch signaling. **(A)** A positive Hill function (λ > 1) describes transcription in presence of an activator that binds to DNA. When the concentration of transcription factor is high, the Hill function relaxes to a constant larger than 1. Conversely, for transcriptional inhibition, a negative Hill function (λ < 1) relaxes to a constant smaller than 1. **(B)** Models of Notch signaling dynamics integrate intracellular signaling (indicated by the blue network and interconnections) with ligand-receptor binding. **(C)** In a lattice model, cells are arranged in a fixed grid. Each position in the grid is identified as a cell, and ligands and receptors belonging to neighboring cells can bind. **(D)** In an agent-based model, space is divided into small fixed regions, and a cell is described by a set of contiguous space regions with the same cell identity (represented here as the color). **(E)** In an off-lattice model, a cell is described by the position coordinates of a set of membrane points. Membrane points of a cell are connected, for instance with elastic springs (continuous black lines). Cell-cell junctions are modeled as binds between pairs of membrane points of neighboring cells (dashed black lines).

In this expression, *K*_*0*_ is the basal transcription rate in absence of NICD, *S*_*0*_ is a threshold concentration of NICD, λ is a fold-change and *n* is a coefficient that regulates how steeply transcription changes as a function of NICD. At low NICD (NICD≪*S*_0_), there is only constitutive production (*K*_prod_ = *K*_0_). Conversely, at high NICD (NICD≫*S*_0_), the transcription rate is scaled by a fold-change (*K*_prod_ = *K*_0_λ). Therefore, λ < 1 implies a decrease of transcription rate (inhibition), while λ > 1 (activation) implies an increase of transcription rate (Figure 2A). Depending on the model, slightly different mathematical definitions might be found for this function.

Γ_Degr_ generically represents a loss term. Loss due to molecule degradation and dilution is usually modeled with a linear function Γ_Degr_ = γ*X*, where γ is the inverse half-life of *X*. In the specific case of Notch signaling, intracellular signaling is coupled with ligand-receptor binding (Figure 2B). Therefore, *X* can represents a receptor or ligand that binds to another ligand/receptor and degrades after NICD release. This is often modeled with a chemical reaction term, thus Γ_Degr_ = *kXY* + γ*X*, where *Y* represents the concentration (or copy number) of a ligand or receptor that binds to *X*, and *k* is the ligand-receptor binding rate constant.

Therefore, a network of *N* interacting biochemical species or genes, such as the intracellular signaling network sketched in Figure 2B, can be described by a collection of variables (*X*_*1*_,*X*_*2*_, …, *X*_*N*_) and a set of *N* ODEs of the form of Eq. 1. In such system of equations, the production term for *X*_*i*_ ($K_{\text{prod}}^{\left(i\right)}$) describes the regulation on *X*_*i*_ due to interactions with all other species in the network.

It is worth mentioning that biochemical and gene regulatory networks are sometimes modeled with Boolean, rather than continuous, variables. A Boolean variable can only assume two states *X* = 0, 1 corresponding to an inactive or active chemical species/gene, respectively. At any given time, the state of a variable (*X*) is determined by the incoming signal from all other chemical species/genes that interact with *X*.

These models of intracellular dynamics can be generalized to a multicellular scenario by arranging cells in a discrete lattice (Figure 2C). In these models, the intracellular signaling dynamics is still described by a set of ODEs. In the specific case of Notch signaling, the biochemical circuits within each cell are coupled by ligands-receptors binding between neighbors (see again Figure 2B). These lattices can have different geometries (square, hexagonal) or can be disordered to study the effect of cell size and shape (Formosa-Jordan and Ibãnes, 2009; Boareto et al., 2015a; Shaya et al., 2017).

Lattice model, however, assume a rigid arrangement of cells on a grid, and cannot take into account biophysical processes including cell migration and cell growth. Biochemical signaling and cell-level behavior can be integrated in agent-based models (Figure 2D). In agent-based models, space is discretized into small volumes, and each cell is represented by the collection of grid points sharing the same kind. This modeling approach has been successfully applied, for instance, in the context of sprouting angiogenesis where cells modify their morphology and migrate to give rise to new blood vessels (Bentley et al., 2008).

Finally, a second possibility to couple biochemical signaling with cell-level dynamics is provided by off-lattice models (Figure 2E). In off-lattice models, the membrane of a cell is described by a set of *N* points connected together according to a pre-defined rule, such as elastic springs (Du et al., 2015). Therefore, the motion of these connected membrane points defines the volume occupied by a cell. In the context of Notch signaling, off-lattice model must further include ligand-receptor binding between neighbors. Stopka et al. (2019) recently developed an off-lattice, multicell model of Notch signaling where membrane points of neighboring cells share adhesion junctions (modeled as elastic springs). Therefore, the number of shared junctions between neighbors modulates the amount of signaling between cells (Stopka et al., 2019).

In both agent-based and off lattice models, the signaling dynamics within each cell can still be described by a set of ODEs. One important difference is that “static” lattice models assume fixed cell volumes; therefore, molecule concentration and copy number are equivalent descriptions. Conversely, Agent-based and off-lattice models allow changes in cell volume, thus requiring adjustment of molecular concentrations.

## Spatiotemporal Patterning Guided by Notch Signaling

In this section, we review experimental systems that exemplify two well-known patterning mechanisms enabled by Notch signaling: lateral inhibition and lateral induction. While lateral inhibition promotes opposite cell fates via biochemical negative feedbacks between the Notch receptor and Delta ligands, lateral induction promotes similar cell fates by positive feedback between Notch and Jagged ligands. Moreover, we review mathematical models that elucidate these patterning mechanisms on idealized, ordered lattices. Experiments and theoretical models help decoding the emergent outcomes of interactions between lateral inhibition and lateral induction mechanisms; specifically, we examine three biological processes that exhibit various degrees of patterning: angiogenesis, inner ear development and epithelial-mesenchymal transition in cancer metastasis. Lastly, we discuss temporal oscillations of Notch observed during somitogenesis as an example of spatiotemporal patterning.

### Biochemical Mechanisms of Lateral Inhibition and Lateral Induction

**FIGURE 3:**
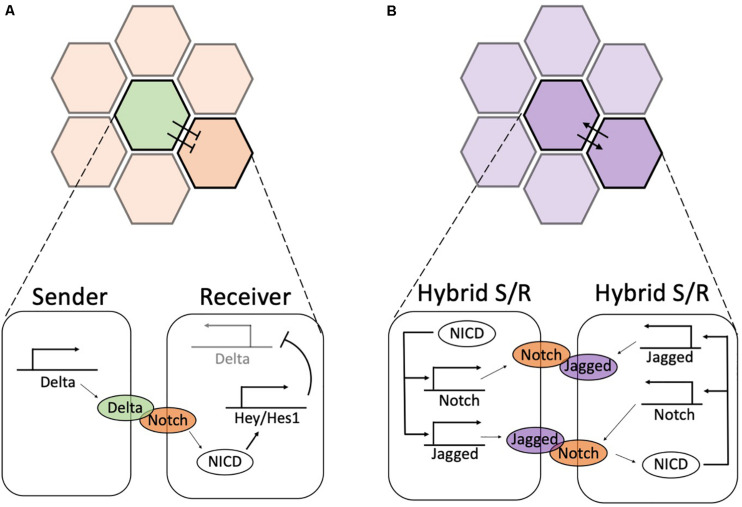
Biochemical feedbacks give rise to lateral inhibition and lateral induction. **(A)** During lateral inhibition, a high-expressing Delta Sender cell (green) suppresses the expression of Delta in its neighbors, hence enforcing a (low-Delta, high-Notch) or Receiver state. Detailed circuit on bottom: Delta ligands of the Sender cell activate Notch receptors in the Receiver. The released NICD activates Hey/Hes1, which in turn suppresses the production of Delta (pointed by the light shading of Delta promoter). Conversely, Notch receptors are not activated in the Sender cells; thus, Delta is freely expressed. **(B)** During lateral induction, neighboring cells mutually promote a similar hybrid Sender/Receiver state. Detailed circuit on bottom: upon activation of Notch receptors, NICD transcriptionally activates Notch and Jagged, hence establishing a high Notch, high Jagged hybrid Sender/Receiver state. In both panels, the color shading in the top highlights the two cells shown in the detailed circuit in the bottom.

Historically, Notch signaling has been first characterized in *Drosophila melanogaster* as a mechanism that induces opposite cell fates among nearest neighbors (Heitzler and Simpson, 1991; Celis and de Garcia-Bellido, 1994; Celis and de Bray, 1997; Huppert et al., 1997; Simpson, 1997; Buceta et al., 2007). The establishment of divergent phenotypes among two neighboring cells, or lateral inhibition, relies on binding of the Notch receptor to ligands of the Delta-like family (Delta in Drosophila; Dll1, Dll3 and Dll4 in mammals – see Table 1) presented at the cell surface of a neighboring cell (Bray, 2006; Andersson et al., 2011). Upon engaging of Delta with the transmembrane Notch receptor, the intracellular domain of Notch (NICD) is cleaved by enzymes and translocates to the cell nucleus. Here, NICD activates Hey/Hes1, which in turn inhibits Delta (Shimojo et al., 2011; Bray, 2016; Sjöqvist and Andersson, 2019; Figure 3A). This negative feedback amplifies small initial differences in ligand and receptor concentrations among nearly equivalent neighbors to establish opposite cell states. The cell with higher levels of Delta can more effectively inhibit Delta in its neighbor, hence assuming a (low Notch, high Delta) or Sender phenotype, while forcing the neighbor to an opposite (high Notch, low Delta) or Receiver phenotype (Collier et al., 1996; Shaya and Sprinzak, 2011) (the green and orange cells in Figure 3A). This basic principle of differentiation regulates cell fate in several developmental and physiological processes. Interesting examples besides Drosophila’s development include angiogenesis (Benedito et al., 2009; Benedito and Hellström, 2013), spinal cord patterning in zebrafish (Appel and Eisen, 1998; Givan et al., 2001; Huang et al., 2012), development of neuroblast cells in early neurogenesis (Skeath and Carroll, 1992; Campos-Ortega, 1993; Homem and Knoblich, 2012), and vulval development in *C. elegans* (Fisher et al., 2007; Louisa et al., 2020). Thus, the Notch-Delta system can be regarded as a two-cell ‘toggle switch’ (Gardner et al., 2000) that enables opposite cell fates and possible switching among them under the influence of biological noise.

Despite being initially characterized as a driver of cell differentiation, Notch signaling can induce a convergent cell phenotype among neighbors through lateral induction (Bray, 2016; Sjöqvist and Andersson, 2019). A positive biochemical feedback between the Notch receptor and ligands of the Jagged/Serrate family establishes similar cell phenotypes that are spatially propagated to neighbors during the development of the inner ear (Lewis et al., 1998; Kiernan et al., 2001, 2006) and vascular smooth muscle cell (Manderfield et al., 2012). The Jagged family in mammals includes two paralogs (Jag1, Jag2), while Drosophila presents a single Serrate subtype (Bray, 2016; Sjöqvist and Andersson, 2019; see Table 1). Ligands of the Jagged/Serrate family are directly activated by NICD (Manderfield et al., 2012). Therefore, Notch-Jagged signaling between neighbors activates a positive feedback that establishes phenotypes with (high Notch, high Jagged) (the purple cells in Figure 3B), occasionally referred to as hybrid Sender/Receiver phenotypes to highlight that both cells send and receive signals (Boareto et al., 2015a). Unless otherwise stated, green and orange colors denote high-Delta (Sender) and high-Notch (Receiver) phenotypes, respectively. Conversely, purple coloring indicates high-Jagged (hybrid Sender/Receiver) cells.

It is important to stress that positive and negative biochemical feedbacks that minimize or amplify initial differences are often assisted by a spatial and/or temporal regulation of Notch ligands and receptors (discussed in more detail by Bray, 2016). For instance, in the development of the *D. melanogaster* wing imaginal disc, the ligand Serrate is expressed only by cells on the dorsal side due to spatial confinement of the upstream transcription factor Apterous (Kim et al., 1995). This sharp boundary creates a stripe of Notch-active cells on the ventral side that leads to tissue growth thereafter (Kim et al., 1995).

### Theoretical Exploration of the Notch-Delta-Jagged Circuit

**FIGURE 4:**
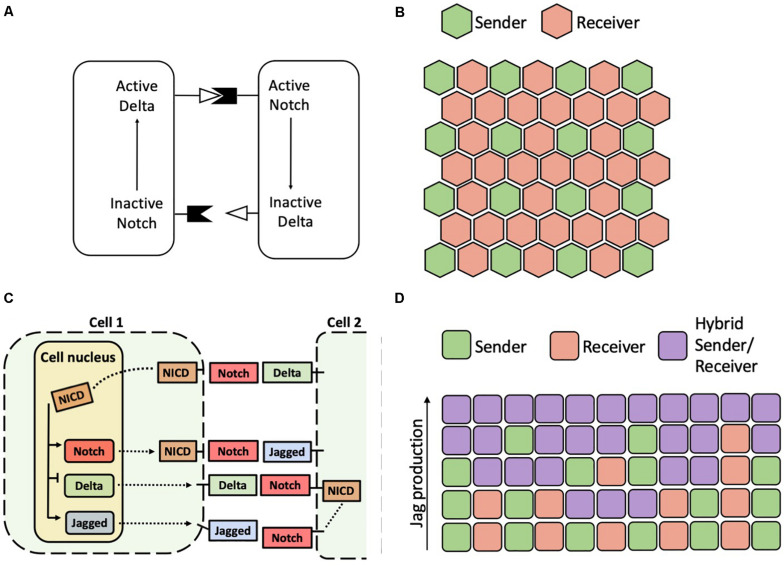
Patterns predicted by models of Notch-Delta and Notch-Delta-Jagged signaling. **(A)** Schematic of the Notch-Delta cell-cell signaling model proposed by Collier et al. (1996). **(B)** A typical solution of the model of Collier and collaborators on a hexagonal lattice with Senders (green) surrounded by Receivers (red). **(C)** Model of the Notch-Delta-Jagged circuit proposed by Boareto et al. (2015a). Solid black arrows in the cell nucleus indicate transcriptional action of NICD. Dashed black lines indicate transport of Notch, Delta and Jagged molecules to cell surface, where they can bind to ligands and receptors of a neighbor cell. **(D)** In a model of the Notch-Delta-Jagged circuit, increasing the cellular production rate of Jagged destabilizes an alternate pattern of Senders and Receivers in favor of a homogeneous array of hybrid Sender/Receiver. Each row represents the pattern on a different one-dimensional chain of cells with increasing production rate of Jagged. Chains of cells with low production of Jagged show an alternation of Senders and Receivers, while chains with higher Jagged production rates show progressively more hybrid Sender/Receiver cells.

Over the last two decades, theoretical models helped understanding the biochemical dynamics leading to lateral inhibition and lateral induction as well as the consequences of these signaling modes at the cell population level. In the first model of Notch-Delta lateral inhibition, Collier et al. (1996) hypothesized that activation of Delta stimulates Notch in the neighboring cells, while activation of Notch restricts Delta within the same cell (Figure 4A). In this model, the homogeneous state where neighbors express the same levels of Notch and Delta is stable for weak biochemical feedback, while cells differentiate into a Sender and a Receiver for strong feedbacks (Collier et al., 1996). When generalized to a spatial distribution of cells, cells tend to arrange in a ‘salt-and-pepper’ pattern where Senders are surrounded by Receivers and *vice versa* (Collier et al., 1996). Therefore, cell patterning in the model depends on the geometric arrangement of cells. While Senders and Receivers can perfectly alternate on a square lattice, patterns on hexagonal lattices typically feature Senders surrounded by six Receivers, hence leading to a 3-to-1 Receiver/Sender ratio (Figure 4B). This patterning arises because contacts between Senders represent a more pronounced instability (Teomy et al., 2019). While contacts between Receiver cells results in the absence of signaling, two Sender cells dynamically compete until one of them eventually become a Receiver (Teomy et al., 2019). This arrangement is well reflected, for example, in the avian inner ear, where high-Delta hair cells are completely surrounded by low-Delta supporting cells (Goodyear and Richardson, 1997).

Further, some mathematical models have encapsulated the ability of Notch signaling to drive both divergent and convergent cell fates. A model developed by Boareto and colleagues considers the transcriptional activity of NICD that inhibits Delta and activates Jagged (Figure 4C). In this simplified representation, Delta and Jagged generically represent the two classes of ligands (Boareto et al., 2015a). In this model, the positive feedback between Notch and Jagged can drive the cells away from lateral inhibition, instead promoting a convergent hybrid Sender/Receiver state. Therefore, if the relative contribution of Notch-Delta signaling is large as compared to that of Notch-Jagged, two neighboring cells fall into lateral inhibition. If Notch-Jagged signaling is dominant, however, the cells fall into the convergent ‘hybrid Sender/Receiver’ configuration with similarly high levels of Notch and Jagged (Boareto et al., 2015a). Therefore, modulating the balance between Notch-Delta and Notch-Jagged signaling in the model leads to transition between salt-and-pepper patterns and homogeneous patterns (Figure 4D). This trend is reminiscent of the dynamical behavior of an intracellular “toggle switch” coupled with self-activation, where the relative strengths of mutual inhibition and self-activation can drive different cell fates (Jolly et al., 2015).

### Interplay of Lateral Inhibition and Lateral Induction Described by Experiments and Mathematical Models

Despite leading to opposite outcomes, lateral inhibition and lateral induction can take place at consecutive developmental steps, such as during inner ear development. Alternatively, they represent different outcomes that are selected based on signaling cues in the extracellular environment, such as during angiogenesis or tumor progression. In this section, we review experiments and mathematical models that raise interesting questions about the interplay between lateral inhibition and lateral induction in three specific contexts: angiogenesis, inner ear development, and epithelial-mesenchymal transition during cancer metastasis.

#### Angiogenesis

**FIGURE 5:**
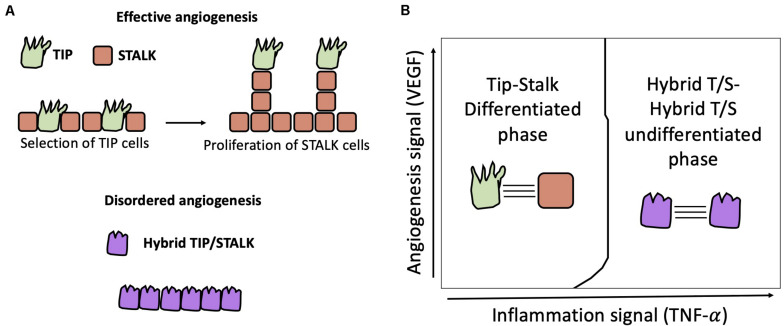
Physiological and pathological angiogenesis. **(A) Top:** physiological angiogenesis is driven by cell differentiation between Tip (i.e., Sender, green) cells and Stalk (i.e., Receiver, orange) cells by Notch-Dll4 signaling. **Bottom:** lack of differentiation can lead to hybrid Tip/Stalk cells (purple) and disordered angiogenesis as seen during tumor development. **(B)** In a model of two cells communicating via Notch-Delta-Jagged signaling, Kang et al. (2019) predicted a transition from Tip-Stalk differentiation to hybrid T/S-hybrid T/S “de-differentiation” triggered by a threshold dose of TNF-α signal that activates Jagged.

Angiogenesis – the growth of new blood vessels from existing ones – is triggered by the hypoxia-induced signal VEGF (Vascular Endothelial Growth Factor). Secreted VEGF molecules bind to VEGF receptors (VEGFR) in the endothelial cells at the boundary of an existing blood vessel (Benedito and Hellström, 2013). Activation of VEGFRs in turn leads to transcriptional activation of the Delta subtype Dll4, hence inducing differentiation between a tip cell with high Dll4, and a stalk cells with low Dll4 by lateral inhibition (Holger et al., 2003; Benedito and Hellström, 2013). Subsequently, tip cells develop filopodia and migrate toward the VEGF gradient, while stalk cells proliferate to support the formation of the new vessel (Figure 5A, top).

Mathematical models suggest that tip-stalk differentiation can be understood as an example of lateral inhibition where external VEGF inputs activate Notch-Dll4 signaling (Bentley et al., 2008; Katie et al., 2014). Moreover, computational models suggest that tip-stalk selection is highly kinetic, and the typical timescale to commit to a specific cell fate varies considerably based on conditions in the extracellular environment as well as intracellular signaling dynamics (Venkatraman et al., 2016).

A binary model of Notch-Delta driven tip-stalk differentiation, however, cannot fully explain some experimental observations (Benedito and Hellström, 2013). For instance, Dll4 can occasionally act as a brake on sprouting angiogenesis by inhibiting endothelial tip formation (Suchting et al., 2007). Conversely, Jagged1 – that usually promotes lateral induction – assists vessel development in mouse models where Notch-Dll4 signaling is antagonized by the glycosylation of Notch by Fringe (Benedito et al., 2009). In addition, lateral inhibition typically leads to patterns with alternate cell fates, while tip cells are typically separated by more than one stalk cell (Benedito et al., 2009).

Various models have been developed to explain deviations from classical Notch-Delta driven angiogenesis. Venkatraman et al. (2016) showed that regulators of Notch signaling such as lunatic fringe can slow down the Tip-Stalk differentiation process, hence giving rise to metastable partial Tip/Stalk states (Venkatraman et al., 2016). To explain sparse patterns where Tips are separated by multiple Stalks, Koon et al., integrated a standard model of Notch-Delta lateral inhibition with intracellular heterogeneity of Notch concentration and tension-dependent binding rate of the Notch-Delta complex (Koon et al., 2018). Interestingly, the addition of intracellular heterogeneity introduces states with intermediate levels of Notch and Delta, thus giving rise to pattern with multiple stalks separating consecutive Tips. Boareto et al. generalized their earlier computational model of the Notch-Delta-Jagged signaling to include VEGF-driven activation of Delta. This model predicts bistability between a Tip phenotype (i.e., Sender) and the Stalk phenotype (i.e., Receiver) when Jagged is weakly expressed (Boareto et al., 2015b). High expression of Jagged, however, stabilizes a homogeneous solution with hybrid Tip/Stalk (i.e., hybrid Sender/Receiver) cells (Figure 5A, bottom). In this model’s interpretation, lateral induction between hybrid Tip/Stalk cells can prevent a binary categorization of migrating and proliferating cells, thus potentially disrupting vessel development (Boareto et al., 2015b).

To elucidate the interplay between Dll4 and Jag1 during angiogenesis experimentally, Kang and colleagues exposed human endothelial cells to both VEGF signal and the pro-inflammatory cytokine Tumor Necrosis Factor (TNF) that activates Jag1 *in vitro* (Kang et al., 2019). Strikingly, the combination of VEGF and low TNF dosage gives rise to longer vessels. At a critical threshold of TNF dosage, however, opposite outcomes (i.e., either robust vessel formation or no vessel formation) were observed in experimental replicates. Finally, TNF dosages above the critical dosage consistently prevented vessel formation (Kang et al., 2019). Mathematical model focusing on the activation of Notch-Delta and Notch-Jagged signaling driven by VEGF and TNF, respectively, suggests a dose-dependent role for Jagged (Kang et al., 2019). While high levels of Jagged can lead to hybrid Tip/Stalk cells and disruption of angiogenesis, low Jagged activity acts synergistically with Delta to refine the alternate pattern of tips and stalks, hence contributing to more robust angiogenesis (Figure 5B). Therefore, increasing TNF dosage can lead to a switch in the role of Jagged from pro-angiogenesis to anti-angiogenesis (Kang et al., 2019).

The dynamics of Tip-Stalk differentiation receives several signaling inputs besides VEGF and TNF. Weinstein et al. (2017) developed a Boolean model of a large regulatory network governing endothelial cell behavior during angiogenesis. This model explores the crosstalk between Notch and several other signaling pathways in the cell as well as the cell microenvironment. It correctly recapitulates the molecular signatures of Tip and Stalk endothelial cells, and offers a platform to integrate signaling crosstalk in a large circuit with the simplification of a Boolean model (Weinstein et al., 2017).

In a pathological context, cancer cells can stimulate the sprouting of new blood vessels in the tumor microenvironment to supplement tumor growth (Kerbel, 2008; Weis and Cheresh, 2011). Typically, tumors exhibit irregular vascular networks that prevent efficient drug delivery (Koganehira et al., 2003; Jain, 2005), and even facilitate passive metastasis by engulfing cancer cells (Bockhorn et al., 2007; Fang et al., 2015). The ability of cancer to induce vasculature makes tumor angiogenesis a potential therapeutic target to halt tumor progression. Strikingly, antitumor drugs that target Dll4, however, do not reduce tumor angiogenesis overall. Instead, anti-Dll4 drugs may result in a higher number of newly formed blood vessels with reduced functionality and chaotic architecture (Kerbel, 2008). Lateral induction of the hybrid tip/stalk phenotype has been proposed as a potential explanation to this paradoxical finding. As anti-Dll4 drugs tilt the balance toward Notch-Jagged signaling, the lack of tip-stalk differentiation amplifies promiscuous cell differentiation and leaky angiogenesis (Boareto et al., 2015b).

As we gain a better understanding of the complex spatiotemporal dynamics of normal and tumor angiogenesis, the advantages and disadvantages of combining drugs targeting angiogenesis with other standard-of-care therapies demand further investigation. Limited exposure to vasculature potentially protects the tumor from therapeutic agents that directly target cancer cells. Thus, perhaps counterintuitively, a transient renormalization of the tumor vasculature, timely synchronized with antitumor drugs, could serve as a potential strategy to alleviate tumor progression (Thurston et al., 2007).

Due to the strong coupling between signaling and cell mechanics observed during angiogenesis, several mathematical models have explored the connection between molecular mechanisms and cell- and organ-level behaviors. Further information on these models, which are not discussed here, are reviewed by Qutub et al. (2009).

#### Inner Ear Development

**FIGURE 6:**
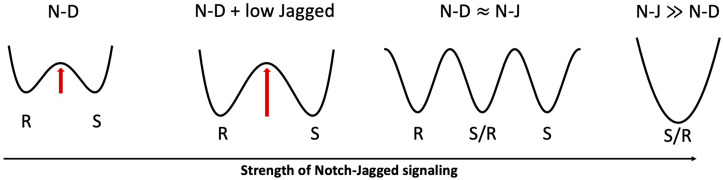
Proposed role of Jagged dosage in Notch-driven cell fate. From left to right: in absence of Jagged (N-D), Sender and Receiver are the only accessible states in an abstract phenotypic landscape; a low Jagged dosage (N-D + low Jagged) increases the stability of Sender and Receiver states (indicated by the higher barrier in the landscape), as seen in inner ear development and angiogenesis; when both Notch-Delta and Notch-Jagged signaling are active (N-D≈N-J), a third hybrid Sender/Receiver state becomes accessible; an overwhelmingly strong Notch-Jagged signaling (N-J≫N-D) stabilizes the hybrid Sender/Receiver as the only accessible state.

Lateral induction and lateral inhibition operate progressively at different stages of the inner ear development to turn an initially homogeneous population of non-sensory cells into a refined mosaic of cells with specific phenotypes. The inner ear is composed of hair cells that convert external stimuli into electrical signals, and supporting cells that provide tissue scaffolding, maintain a stable electrochemical environment, and occasionally differentiate to replenish the hair cell population after an injury (Kiernan et al., 2005; Neves et al., 2013). During the prosensory cell specification phase, Notch activates Jag1, which in turn sustains Notch in prosensory cells via lateral induction (Eddison et al., 2002; Daudet and Lewis, 2005; Daudet et al., 2007). Thus, the activation of Notch and Jag1 not only establishes the hair cell phenotype, but also propagates it through lateral induction up to several cell diameters (Hartman et al., 2010). Later, in the hair cell differentiation phase, Notch-Dll1 signaling establishes the final pattern where hair cells (i.e., the Senders) are surrounded by supporting cells (i.e., the Receivers) (Eddison et al., 2002; Daudet and Lewis, 2005; Daudet et al., 2007). For further insights on the role of Notch signaling in the inner ear development, a thorough review is offered by Neves et al. (2013). Interestingly, Petrovic et al. (2014) argued with experiments and mathematical modeling that Jag1 acts synergistically with Dll1 during the hair cell differentiation phase in enforcing a robust lateral inhibition by acting as a competitive inhibitor for Dll1. Similar to the model of Notch-driven angiogenesis proposed by Kang et al. (2019), a dose-dependent role for Jagged is suggested in inner ear development. While high levels of Jagged lead to a homogeneous state where cells attain a hybrid Sender/Receiver fate, a weak expression of Jagged can act synergistically with Dll1 to refine the alternate pattern of Sender and Receivers. In the presence of a dominant Notch-Delta signaling, additional Jagged tends to compete with Delta over binding Notch receptors, resulting in a greater activation of NICD, and thus suppression of Delta, in Receiver cells (Petrovic et al., 2014). In this case, the ability of Jag1 to establish a convergent cell fate is negligible as compared to the cell differentiation promoted by Delta. When the signaling through the Notch-Jagged “branch” of the pathway becomes too strong, however, lateral induction dominates the patterning (Figure 6). Interestingly, the dose-dependent role of Jagged is only observed in mathematical models of extended two-dimensional lattices. For instance, Boareto et al. (2015a) showed that Notch-Delta signaling robustly give rise to salt-and-pepper patterns of Sender and Receivers on a one-dimensional chain (see Figure 4D again). In the two-dimensional lattice cells have a higher number of nearest neighbors – and thus potentially contradictory external inputs to process – hence increasing the probability of mistakes, or Sender-Sender contacts, in the pattern.

#### Epithelial-Mesenchymal Transition and Cancer Metastasis

Metastases represents the most critical step during tumor progression. Typically, cancer cells invade the circulatory system, reach anatomically distant sites and give rise to a secondary tumor (Gupta and Massagué, 2006). These cells can migrate individually as well as collectively as multi-cellular clusters with varying sizes depending on cancer type, stage and patient individualities (Cheung and Ewald, 2016; Jolly et al., 2017; Bocci et al., 2019b).

**FIGURE 7:**
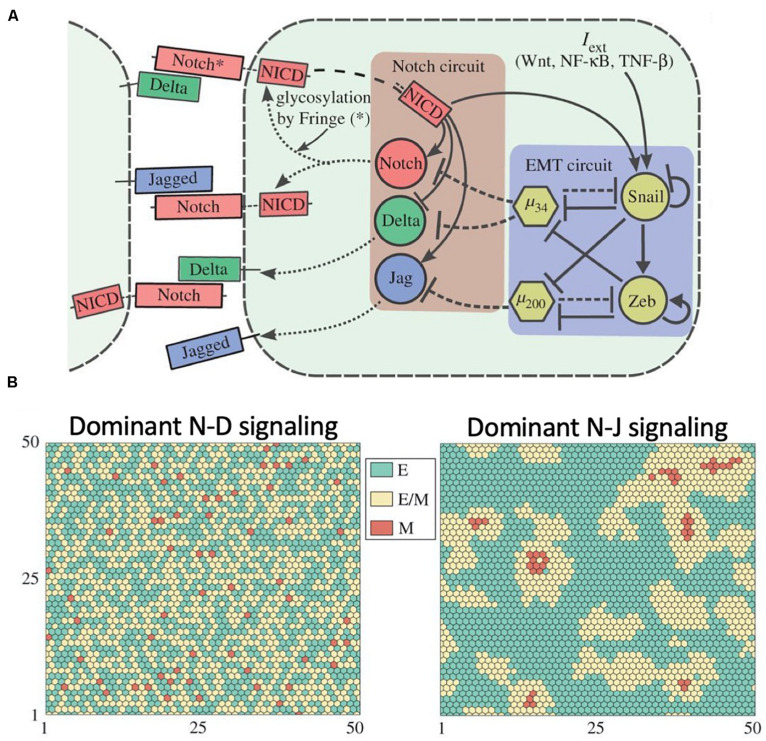
Role of Notch signaling during Epithelial-Mesenchymal Transition. **(A)** Proposed coupling between the Notch-Delta-Jagged circuit and the core EMT regulatory network proposed by Boareto et al. (2016). **(B)** Mathematical modeling of the Notch-EMT circuit predicts patterns where hybrid epithelial/mesenchymal and mesenchymal cells are mostly surrounded by epithelial cells in presence of dominant Notch-Delta signaling (left) and patterns with clusters of hybrid E/M cells in presence of dominant Notch-Jagged signaling (right). In this figure, green, yellow and red represent epithelial, hybrid epithelial/mesenchymal and mesenchymal cells, respectively. The figure is adapted from Boareto and collaborators with permission from the published under a creative common license (Boareto et al., 2016).

Generally, epithelial cancer cells partially or completely lose their cell-cell adhesion and acquire motility by undergoing the epithelial-mesenchymal transition (EMT) (Nieto et al., 2016). EMT can be activated by signaling cues in the tumor microenvironment in a cell autonomous manner as well as by Notch signaling. Activation of Notch signaling can be suppressed by EMT-inhibiting microRNAs such as miR-34 and miR-200 (Brabletz et al., 2011; de Antonellis et al., 2011; Bu et al., 2013; Bocci et al., 2019d). Notch signaling, however, can induce EMT by activating the EMT-inducing transcription factor SNAIL (Niessen et al., 2008; Sahlgren et al., 2008; Figure 7A).

An effort to elucidate the coupled dynamics of Notch signaling with the EMT gene regulatory network (Boareto et al., 2016) suggests that Delta-driven and Jagged-driven EMT can have different consequences at the level of multi-cellular patterning in a cancer tissue. While cells undergoing Notch-Delta-driven EMT are typically surrounded by epithelial cells, Notch-Jagged-driven EMT enables clustering among cells undergoing EMT (Figure 7B), hence potentially facilitating the formation of migrating multi-cellular cohorts in a tissue (Boareto et al., 2016).

Besides, Jag1 can also stabilize a hybrid epithelial/mesenchymal (E/M) cell phenotype (Boareto et al., 2016). Such hybrid E/M phenotype(s) can partially maintain cell-cell adhesion while gaining motility, and can invade as circulating tumor cell clusters (CTC clusters) that have elevated metastatic potential (Cheung and Ewald, 2016; Pastushenko and Blanpain, 2018; Sha et al., 2018; Jia D. et al., 2019). Experimental observations support this proposed role of Notch-Jagged signaling, although mostly through indirect evidence. First, CTC clusters from patients have a high expression of Jagged and co-express epithelial and mesenchymal markers, indicative of a hybrid epithelial/mesenchymal phenotype (Aceto et al., 2014; Jolly et al., 2017). Conversely, single CTCs mostly lack Jagged expression (Jolly et al., 2017). Second, Jag1 was identified as among top 5 differentially expressed genes in cells positive for K14, a marker for cluster-based migration (Cheung et al., 2013). Generalizations of this framework identified additional biochemical pathways that act as “phenotypic stability factors” (PSFs) and stabilize hybrid E/M phenotype by coupling to the core Notch-EMT circuit. Examples include NUMB, NF-kB and IL-6 (Bocci et al., 2017, 2019a). Consistently, overexpression of PSFs such as NUMB correlates with a worse patient survival in various cancer types (Jia et al., 2015; Bocci et al., 2017, 2019a,e). Recently, the epigenetic landscape and transition dynamics during EMT have been unraveled with a stochastic dynamical modeling approach (Li et al., 2016; Li and Balazsi, 2018; Jia W. et al., 2019). These models suggest the presence of multiple intermediate hybrid E/M states and indicate plausible transition routes between EMT phenotypes in the noisy cellular epigenetic landscape (Li et al., 2016; Li and Balazsi, 2018). Certainly, understanding how Notch signaling affects the stability and transitions between EMT phenotypes from a landscape perspective is an exciting future direction for theoretical modeling.

To metastasize, migrating cancer cells need the proliferation potential and resistance to therapies typical of cancer stem cells (CSCs). Typically, cells undergoing a partial or complete EMT also show traits of CSCs (Mani et al., 2008; Grosse-Wilde et al., 2015; Pastushenko et al., 2018; Bocci et al., 2019c). Mathematical modeling of the gene regulatory networks underlying EMT, Notch and stemness suggests that Notch-Jagged signaling can promote a “window of opportunity” where cancer cells exist in a hybrid E/M, stem-like phenotype with aggravated metastatic potential (Bocci et al., 2018a; Nie, 2018). Consistent with this prediction, CSCs display enhanced levels of Notch and Jagged across several cancer types including glioblastoma, pancreatic cancer, colon cancer and breast cancer (Wang et al., 2009; Sikandar et al., 2010; Zhu et al., 2011; Yamamoto et al., 2013). Moreover, the glycosyltransferase Fringe which promotes Notch-Delta interactions over Notch-Jagged is reported as a tumor suppressor in multiple cancers (Xu et al., 2012; Yi et al., 2013; Zhang et al., 2014). Furthermore, it was recently shown *in vitro* that knockdown of Jag1 inhibits the formation of tumor emboli in hybrid E/M inflammatory breast cancer (IBC) – a rare but highly aggressive form of breast cancer that moves largely collectively through clusters (Jolly et al., 2017) – cells SUM149 (Bocci et al., 2019a).

Notch signaling can also regulate spatiotemporal pattern formation at the level of a tumor tissue. Analysis of breast cancer tissues highlighted subsets of mesenchymal CSCs at the tumor invasive edge, while subsets of hybrid E/M CSCs were largely localized in tumor interior (Liu et al., 2014). A recent computational model developed by Bocci et al. suggests that Notch-Jagged signaling may contribute to generating this spatial heterogeneity. In the presence of a diffusive EMT-inducing signal such as TGF-β, Notch-Jagged signaling, but not Notch-Delta signaling, can give rise to large populations of CSCs. CSCs subsets at the tumor invasive edge are highly exposed to EMT-inducing signals and have a higher likelihood of undergoing EMT, whereas CSCs in the tumor interior are less exposed to EMT-inducing signals and hence retain a hybrid E/M phenotype (Bocci et al., 2019a). Given the varying metabolic profiles of these CSC subsets (Luo et al., 2018), such patterning is reminiscent of spatial self-organization of metabolically diverse phenotypes in other contexts such as bacterial colonies (Bocci et al., 2018b; Varahan et al., 2019).

Finally, the transition to a mesenchymal phenotype is not exclusive to epithelial cells. Besides undergoing tip-stalk differentiation in sprouting angiogenesis, endothelial cells can alternatively undergo Endothelial-to-Mesenchymal Transition (EndMT) (Lamouille et al., 2014). While tip-stalk differentiation maintains cell-cell adhesion, EndMT leads to the detachment of endothelial cells. The underlying circuitry associated with these different transition routes involves Notch, the EMT network, and other pathways such as HIF1-alpha and TGF. Recently, this large circuit has been modeled as a Boolean network, offering suggestions about the specific signaling features that distinguish the two transitions (Weinstein et al., 2020).

### Oscillations and Synchronization as Seen in the Somite Segmentation Clock

So far, we discussed mechanisms of spatial patterning. Due to its crosstalk with other signaling pathways, however, Notch can exhibit non-trivial temporal patterns. As an example, here we discuss somite segmentation, a well-known example of Notch oscillatory dynamics. During somite segmentation, the embryo’s body axis is segmented into somites – blocks of epithelial cells that later give rise to vertebrae and tissues in the adult body (Andrew et al., 2012). Segmentation is organized by a precise spatiotemporal clock. Traveling waves of gene expression move along the body axis and stop at the location of a following segmentation event (Andrew et al., 2012).

**FIGURE 8:**
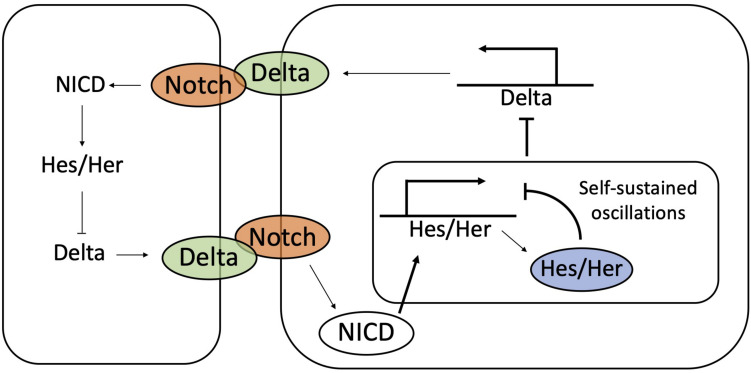
The coupling between Notch-Delta and Hes/Her signaling synchronize temporal oscillations during somitogenesis. Hes/Her can autonomously give rise to sustained oscillations by self-inhibition of Hes/Her protein. The coupling between Hes/Her and Notch-Delta signaling synchronizes oscillations between neighbors.

Oscillations in gene expression are generated in a cell autonomous manner via an autoregulatory negative feedback by Hes/Her proteins. Upon protein productions, Hes/Her molecules dimerize and suppress their own transcription (Lewis, 2003; Monk, 2003). The delay between transcription and protein synthesis gives rise to oscillations in Hes/Her gene expression (Figure 8) with a period of about 2–3 h (Hirata et al., 2002; Shimojo et al., 2008). This model, however, is not sufficient to explain how oscillations maintain a precise cell to cell synchronization in time and space.

Several experimental observations suggest a role for Notch-Delta signaling in synchronizing oscillations in neighboring cells, due to the biochemical coupling between the Notch and Hes/Her pathways. As previously discussed, NICD transcriptionally activates the family of Hes/Her molecules, which in turn, represses Delta (Shimojo et al., 2011; Bray, 2016; Sjöqvist and Andersson, 2019). Therefore, self-sustained oscillations of Hes/Her can potentially propagate to Notch (Figure 8). Zebrafish models indicate a periodic expression of Delta ligands during somite segmentation (Jiang et al., 2000), while mouse models show oscillations of Notch, Delta and NICD (Huppert et al., 2005; Bone et al., 2014; Shimojo et al., 2016).

Notch-Delta binding potentially provides information about the phase of the Hes/Her clock in neighbors. Mathematical modeling of the Notch-Hes/Her circuit developed by Lewis and colleagues (Lewis, 2003; OZbudak and Lewis, 2008) suggests that (i) oscillation can be self-sustained by the autoregulatory Hes/Her feedback loop, but (ii) Notch-Delta progressively couples and eventually synchronizes the clocks of neighboring cells (Lewis, 2003; OZbudak and Lewis, 2008). In other words, each cell can be viewed as an independent biochemical oscillator, and the exchange of ligands through the Notch receptor synchronizes the oscillations of the different cells (Shimojo and Kageyama, 2016; Figure 8). This model is supported by observation in Zebrafish mutants that do not express Notch and Delta. In these mutants, segmentation is defective, and cells are arranged in heterogeneous patterns of high Hes/Her and low Hes/Her indicative of asynchrony in the cell population (Riedel-Kruse et al., 2007; Delaune et al., 2012).

It remains unclear whether Notch’s unique role is to ensure robust temporal correlation among neighbors. While it is generally accepted that Hes/Her self-inhibition is sufficient to generate temporal patterns, a number of studies in mouse models suggest that Notch might be required for oscillations. For further details, a comprehensive review on the role of Notch signaling in the somite segmentation clock is offered by Venzin and Oates (2019).

## Non-Canonical Modulation of Notch Signaling

In the previous section, we discussed mechanisms of lateral inhibition and lateral induction guided by biochemical feedbacks between Notch and its ligands. In this section, we review mechanisms that modulate Notch signaling besides canonical positive and negative transcriptional feedbacks. These include dependence on cell-cell contact area and cell packing geometry, binding between receptors and ligands within the same cell, specificity in the affinity between receptor and ligand paralogs, and mechanisms enabling signaling beyond nearest neighbor. From a phenomenological standpoint, these mechanisms can be viewed as additional features beyond the simple nearest neighbor signaling mechanism.

### Variability of Cell Packing and Contact Area

In the previous section, we developed a geometrical intuition on lateral inhibition that is based on alternate arrangement of Sender and Receiver cells. Mathematical modeling of Notch-Delta signaling helps understand these patterning dynamics on idealized ordered lattices. For instance, Notch-Delta signaling leads to a very specific pattern where Senders are surrounded by six Receivers on a perfect hexagonal lattice (see Figure 4B). Disordered lattices with variable cell sizes and number of nearest neighbors can lead to deviations from the standard “salt-and-pepper” pattern.

The development of the basilar papilla, the avian equivalent of the mammals’ organ of Corti, exemplifies how fluctuations in cell arrangement modulate lateral inhibition. The fully developed basilar papilla consists of a hexagonal mosaic where Sender cells (i.e., hair cells) are surrounded by six Receiver cells (i.e., supporting cells) (Goodyear and Richardson, 1997). Goodyear and Richardson found experimental evidence of dynamic cell rearrangement in the early development of the basilar papilla in a seminal study (Goodyear and Richardson, 1997). At earlier developmental stages (6–7 days), cell packing in the papilla is irregular and features cells with variable size and shape. Consequently, the number of nearest neighbors fluctuate between 3 and 8 cells (Goodyear and Richardson, 1997). This underdeveloped mosaic allows occasional contacts between hair cells. Later on, cell packing relaxes toward a precise hexagonal mosaic and the “mistakes” in the patterning are corrected (Goodyear and Richardson, 1997).

**FIGURE 9:**
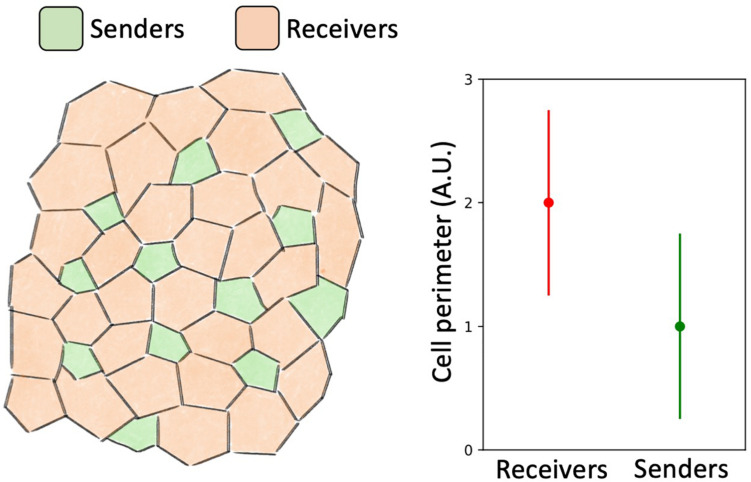
Mathematical modeling predicts a correlation between cell size and fate. Mathematical model of Notch-Delta signaling on a disordered lattice developed by Shaya et al. (2017) suggests that larger cells assume a Receiver phenotype and smaller cells assume a Sender phenotype. **Left:** a typical spatial patterning of Senders (green) and Receivers (red) predicted by mathematical modeling. **Right:** cells with large perimeter tend to become Receivers while cells with smaller perimeter tend to become Senders.

The size of shared contact area between neighbors is expected to fine-tune Notch signaling. Shaya and collaborators investigated the relation between cell size and cell fate by integrating experimental and computational methods (Shaya et al., 2017). By incorporating live-cell imaging reports to track the activity of Notch and Delta, they showed that signaling between pairs of nearest neighbors correlates with their cell-cell contact area. Smaller cells produced Delta at a higher rate and eventually became hair cells, while larger cells generally committed to a non-hair, supporting phenotype (Shaya et al., 2017). This result was reproduced by a mathematical model that generalized the seminal Notch-Delta model of Collier et al. (1996) to a disordered lattice with variable cell size (Shaya et al., 2017; Figure 9). In the simplest model of lateral inhibition, Senders are selected from a homogeneous population by spontaneous breaking of symmetry and amplification of initial differences in protein levels (Collier et al., 1996). Instead, this experiment shows that the fluctuations of cell size contribute to cell fate selection by introducing a weightage factor in the extent of Notch signaling between neighbors (Shaya et al., 2017).

### Cis-Interactions

**FIGURE 10:**
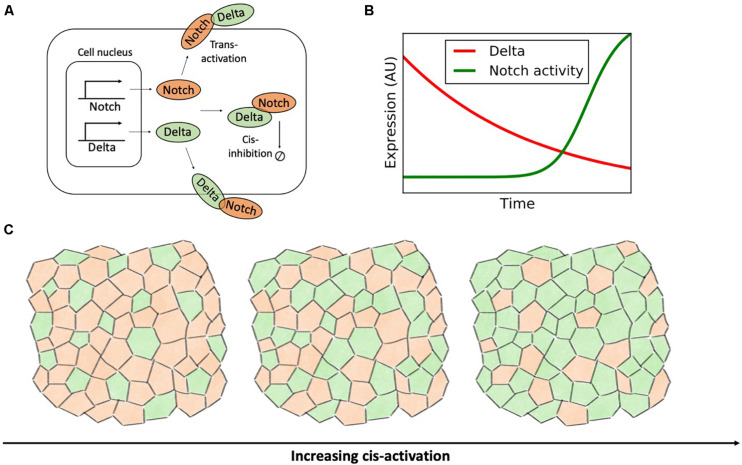
Cis-activation destabilizes the ordered lateral inhibition pattern. **(A)** Binding of Notch and Delta molecules within the same cell leads to the degradation of the receptor-ligand complex without downstream signaling. **(B)** In a time-lapse microscopy experiment by Sprinzak et al. (2010), the concentration of Delta (red) gradually decays exponentially due to dilution and cell division. Conversely, the activity of Notch (green) is turned on sharply when the concentration of Delta decreases below a threshold. This panel is adapted from Sprinzak et al. (2010). **(C)** In a model of Notch-Delta signaling on a disordered lattice developed by Formosa-Jordan and Ibanes (2014), increasing the rate of cis-activation progressively disrupts lateral inhibition patterns. Left: in absence of cis-activation, Notch-Delta signaling gives rise to a pattern where Senders (green) are surrounded by Receivers (orange). For increasing levels of cis-activation, cell fate becomes cell autonomous and the fraction of Senders progressively increases (rightmost plots).

Although Notch has evolved as a cell-cell signaling mechanism, receptors and ligands can bind within the same cell. Ligand-receptor binding within the same cell, or cis-interaction, does not lead to downstream signaling, but rather to ligand-receptor complex degradation (cis-inhibition) (Celis and de Bray, 1997; Micchelli et al., 1997; Del Alamo et al., 2011). Despite not contributing to signaling, cis-inhibition can compete with the canonical Notch pathway by sequestering Notch receptors and ligands (Figure 10A).

Sprinzak et al. (2010) used time-lapse microscopy to evaluate Notch activation in response to external Delta ligands (standard trans-activation) and endogenous Delta (cis-interaction). While Notch receptors trans-activate gradually in response to external Delta, the response to indigenous, cis-Delta is sharp (Figure 10B). Therefore, cis-inhibition silences Notch signaling when the intracellular Delta exceeds a threshold concentration (Sprinzak et al., 2010). This mechanism improves the robustness of lateral inhibition by further inactivating Notch in Sender cells. The authors further employed mathematical modeling to evaluate the behavior of an ensemble of kinetic models of Notch-Delta signaling with randomized parameters. Compared to a control model lacking cis-inhibition, models with cis-interactions yield lateral inhibition over a much broader parameter range by further refining defects in the patterning of Sender and Receiver cells (Sprinzak et al., 2011). The role of cis-inhibition, however, is not just restricted to proof-reading, but can rather be pivotal for cell-fate decision. For instance, loss of cis-inhibition compromises cell fate specification during the development of photoreceptors in Drosophila (Miller et al., 2009).

Although cis-interactions are mostly known to degrade Notch signaling without any contribution to signaling, experiments recently reported cell autonomous activation of Notch, such as in the cases of Drosophila bristle precursor cells and cell cycle regulation in T cells (Coumailleau et al., 2009; Guy et al., 2013). These experiments raise interesting questions about the competition between intracellular and intercellular signaling in modulating cell fate decisions. Nandagopal and colleagues engineered a synthetic system where cells constitutively express Notch while production of Delta is controlled experimentally (Nandagopal et al., 2019). Interestingly, extremal expression of Delta silenced Notch activity, whereas intermediate Delta expression maximized cis-activation (Nandagopal et al., 2019). To rationalize these observations, the authors developed various classes of mathematical models where cis-interactions can lead to either cis-activation or cis-inhibition with different rates. Interestingly, the non-monotonic response of Notch as a function of Delta concentration could only be reproduced by models with higher-order interactions and formation of clusters with multiple ligands and receptors (Nandagopal et al., 2019). Indeed, oligomerization of Notch receptors and ligands has been reported in the Notch pathway (Bardot et al., 2005; Nichols et al., 2007; Nandagopal et al., 2018).

Given the role of cis-inhibition in enforcing robust lateral inhibition, it can be postulated that a switch from cis-inhibition to cis-activation would compromise precise cell patterns of Sender and Receiver cells. Formosa-Jordan and Ibanes (2014) investigated the implication of Notch-Delta cis-activation in a disordered multicellular lattice model with variable cell size and shape. Compared to the mathematical model by Shaya et al. (2017) discussed in the previous section, the authors did not focused explicitly on the correlation between cell size and cell fate, but rather on how cis-activation biases patterns of Senders and Receivers. Their mathematical model confirms that cis-activation prevents robust lateral inhibition and instead introduces disordered patterns (Formosa-Jordan and Ibanes, 2014). Specifically, in presence of strong cis-activation, cell dynamics is predominantly cell-autonomous, rather than driven by nearest neighbors. Hence, cis-activation progressively increase the fraction of high-Delta Sender cells in the lattice model (Figure 10C). Indeed, cis-activation introduces a negative intracellular feedback where Delta ligands in the Sender cell promote their own inhibition by activating Notch receptors, hence driving the system away from the target Sender state with (low Notch, high Delta).

### Specificity in Ligand-Receptor Binding Affinity

The number of Notch receptor and ligand subtypes varies considerably in different species (see Table 1). Typically, mammals have four different paralogs of the Notch receptor (Notch1–4), three Delta-like ligands (Dll1, Dll3, Dll4), and two Jagged ligands (Jag1, Jag2). Although the effect on the receiving cell is identical (i.e., NICD release), interactions through different ligand-receptor pairs can lead to differences in the downstream signaling cascade (Bray, 2016; Sjöqvist and Andersson, 2019).

First, binding affinities depend on the molecular structure. For instance, Notch1 has a greater affinity to Dll4 than to Dll1 and Jag1 (Luca et al., 2017a). Moreover, different ligand-receptor pairings can lead to different dynamical responses in the receiving cell. For instance, Nandagopal and colleagues proposed that Notch1 can dynamically discriminate the ligands Dll1 and Dll4 in mouse and hamster cells (Nandagopal et al., 2018). Namely, while Dll4 activates Notch1 in a sustained manner, Dll1 gives rise to pulses of Notch1 activity (Nandagopal et al., 2018). Differences arise also in the ligand ability to cis-inhibit Notch receptors. For instance, Dll4 but not Dll1, can efficiently cis-inhibit Notch1 in mice cells (Preuße et al., 2015), reminiscent of the greater Notch1-Dll4 affinity observed in trans-activation (Luca et al., 2017a). Moreover, the ligand Dll3 typically does not trans-activate any of the four Notch subtypes but only contributes to cis-inhibition (Ladi et al., 2005; Chapman et al., 2011).

**FIGURE 11:**
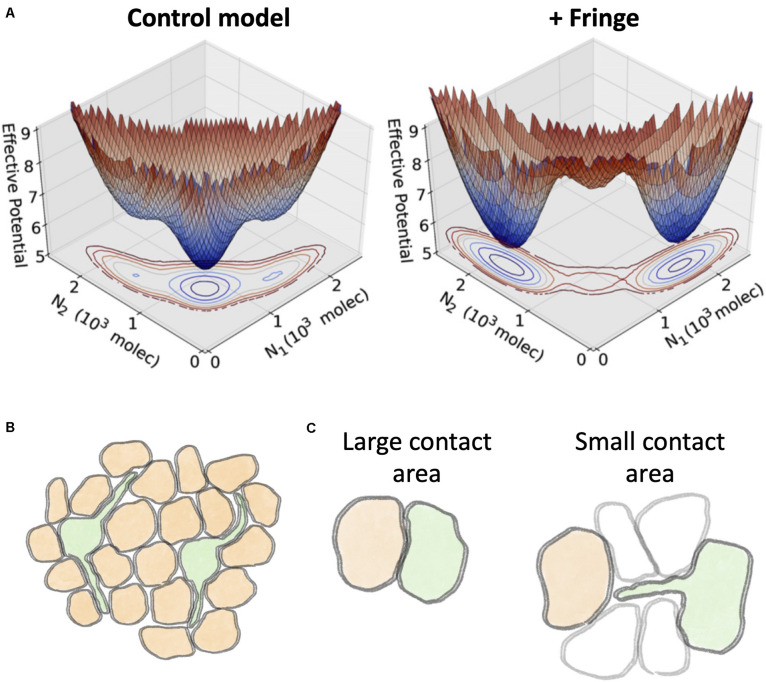
Effect of Fringe glycosylation and filopodia on Notch signaling. **(A)** A mathematical model of Notch-Delta-Jagged signaling by Jolly et al. (2015) predicts a switch in cell fate due to Fringe glycosylation. The effective potential of a two-cell model depicts the probability for the two cells to assume specific levels of Notch (N_1_ and N_2_, respectively). A control model without the effect of Fringe glycosylation (left) exhibits a single dominant minimum where both cells are hybrid Sender/Receiver with same Notch levels. Conversely, a model with Fringe modifies the landscape and introduces two separate states corresponding to Receiver-Sender (N_1_≪N_2_) and Sender-Receiver (N_1_≫N_2_). **(B)** Through filopodia, Sender cells (green) can potentially inhibit the Sender state in cells beyond nearest neighbors. **(C)** Schematic representation of the regimes of Notch-Delta signaling predicted by mathematical modeling by Khait et al. (2016). **Left:** when cells share a large contact area, diffusion of Delta ligands is negligible. **Right:** when the contact area is small, such as in the case of contact through filopodia, the signaling depends crucially on the diffusion of Delta ligands. Panel **(A)** is adapted from Jolly et al., with permission from the published under a creative common license (Jolly et al., 2015).

Mechanisms that modify the binding affinity between the various subtypes of receptor and ligand can potentially result in a shift in cell fate by introducing an asymmetry between Delta and Jagged ligands. One such well-characterized mechanism is the glycosylation by Fringe proteins that results in a conformational change in the extra cellular domain of the Notch receptor (Jane and Wu, 1999; Nadia and Rana, 2011). Glycosylation typically decreases the binding affinity of Notch with Jagged ligands both in trans- and cis-interactions (Hicks et al., 2000; Ladi et al., 2005; Hou et al., 2012; LeBon et al., 2014). Mathematical modeling of the Notch-Delta-Jagged signaling suggests that Fringe can stabilize the Sender and Receiver cell states by restricting the binding between Notch and Jagged, while loss of Fringe may tilt the balance toward Notch-Jagged signaling and lateral induction (Jolly et al., 2015; Figure 11A).

### Interactions Beyond Nearest Neighbor Through Filopodia

Although the Notch pathway is primarily designed as a pairwise signaling mechanism among nearest neighbors, beyond nearest neighbors’ interactions are occasionally enabled by filopodia.

Filopodia can extend up to several cell diameters and thus introduce contacts beyond nearest neighbor (Joussineau et al., 2003; Eom et al., 2015; Huang and Kornberg, 2015; Figure 11B). For instance, in the bristle patterning of Drosophila, the sensory organ precursor cells (SOPs) with high Delta (i.e., the Sender cells) are separated by 4–5 receiver cells. This spacing, much larger than typically observed in lateral inhibition systems, is explained by dynamically rearranging filopodia that can give rise to transient contacts among non-neighbor cells (Cohen et al., 2010). This signaling between cells that are not adjacent to one another has been interpreted as a source of noise that refines the patterning (Cohen et al., 2010).

Filopodia-driven signaling raises questions on how Notch can be effective when cells communicate through a small contact area. Khait et al. (2016) reported that the diffusion coefficient of Dll1 can vary over an order of magnitude (0.003–0.03μm^3^/s) from cell to cell in hamster ovary cells (Khait et al., 2016). Based on this experimental finding, the authors developed a kinetic theoretical model including ligand-receptor binding at cell surface and lateral diffusion of Notch and Delta molecules across the cell surface. This framework highlights opposite regimes of signaling. When the radius of the shared contact area between cells (b) is larger than the typical diffusion length scale (λ), diffusion effects are negligible and the signaling depends on only the contact area. In the opposite regime (λ > *b*), however, the signaling strongly depends on the influx of Delta ligands in the contact area but only weakly on the size of the contact area (Khait et al., 2016; Figure 11C). Diffusion coefficients in filopodia are larger by up to a 10-fold than in bulk membrane, possibly explaining how thin filopodia can still play an important role in Notch signaling (Khait et al., 2016).

## Indications of a Role for Mechanosensitivity in Notch Signaling

Activating Notch signaling requires mechanical pulling on the ligand-receptor complex leading to NICD cleavage. Therefore, the signaling operates optimally within a certain range of mechanical constraints (Meloty-Kapella et al., 2012; Wang and Ha, 2013; Chowdhury et al., 2016). In contexts such as collective epithelial migration and cardiovascular morphogenesis, cells continuously adapt their shape, tensions and stresses. It can be speculated that these biophysical factors add a further layer of regulation on Notch-driven patterning. While the role of mechanosensitivity is more quantitatively understood at the molecular scale of ligand-receptor interaction, its consequences on multicellular patterning are still largely unexplored. The following two sections offer recent evidence suggesting a role for mechanosensitivity in leader-follower differentiation during collective epithelial cell migration and cardiovascular morphogenesis.

### Lateral Inhibition and Mechanics Select Leader and Follower Cells During Collective Epithelial Cell Migration

**FIGURE 12:**
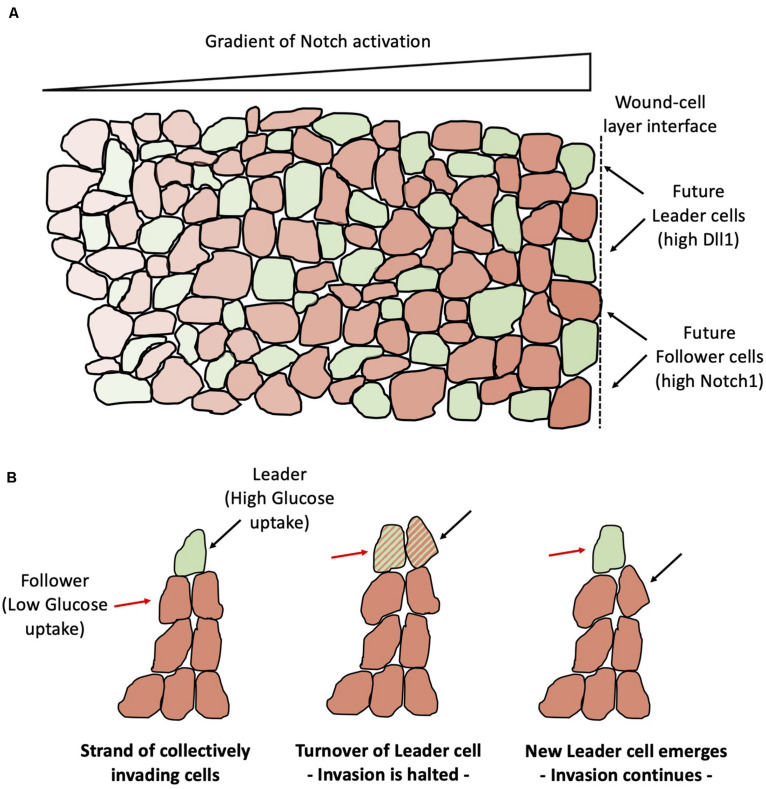
Leader-Follower differentiation and turnover during collective epithelial migration. **(A)** Notch-Dll1 signaling differentiates cells that become leaders of the migration (green) and cells that become followers (orange). Notch-Dll1 signaling is more active toward the wound-cell layer interface (indicated by the black dashed line at the right end) and progressively inactivates far from the interface. **(B)** In strands of cells that migrate collectively, leaders have higher glucose uptake (left). Invasion halts in absence of a clear leader (center). The invasion continues after replacement of the leader cell (right). The red arrow points to a cell that is initially a follower and eventually emerges as the new migration leader. The black arrow points to a leader cell that is later substituted by a new leader.

Collective cell migration is commonly observed in physiological and pathological processes, including morphogenesis, wound healing and cancer metastasis. Collectively migrating cells conserve their cell-cell adhesion through several mechanisms, such as adherens junctions (Friedl and Mayor, 2017; Barriga et al., 2018). Typically, some cells at the front of the migrating cell layer assume a distinct morphology characterized by an enlarged size and ruffling lamellopodia, and are labeled as “leaders” at the migration (Yang et al., 2016). In a typical scratch assay that mimics wound healing, the mechanical injury at the boundary can generate a gradient of activation of several signaling pathways, with the strongest response in cells adjacent to the boundary and gradually decreasing in the inner region (Riahi et al., 2014; Figure 12A). Reminiscent of branching angiogenesis, the differentiation between leader and follower cells is regulated by the Notch-Delta pathway. Specifically, approximately 25% of the cells at the leading edge are leaders with high expression of Dll1. Conversely, cells with low Dll1 and high Notch1 become followers (Riahi et al., 2015). Interestingly, approximately 10% of cells transiently increase Dll1 after wounding but ultimately become followers, showing that the leader-follower differentiation is regulated in a highly dynamical manner by the Notch1-Dll1 pathway (Riahi et al., 2015), similar to the dynamical balance of tip-stalk decision-making in angiogenesis (Jakobsson et al., 2010).

Notably, leader-follower selection depends on feedback loops among Notch signaling and mechanical stresses. Indeed, receptor-ligand binding and the conformational change in the Notch1 domain thereafter require maintaining the receptor-ligand bond for enough time, which might be jeopardized by forces applied to the receptor or ligand (Luca et al., 2017a, b), as can happen in the presence of mechanical injury during wound healing. Mechanical stresses inhibit the expression of Dll1 and prevent the selection of leader cells. Comparing the spatial distribution of mechanical forces and Dll1 expression suggests that the reduction of cellular stress at the boundary allows an effective Notch1-Dll1 signaling and leader-follower selection via lateral inhibition and gives rise to the observed gradient of Notch activation (Riahi et al., 2015). In the classic lateral inhibition scenario, Senders and Receivers are selected by stochastic fluctuations from competing cells that are initially in a similar cell state. A recent experiment showed via monolayer stress microscopy that mechanical interactions among followers cells behind the leading edge determine the selection and emergence of the leader cells at the leading edge (Vishwakarma et al., 2018). In other words, this finding suggests that follower cells decide the leader, not the other way around as has been a long-held belief. Another recent study shows that a leader cell maintains its foremost spatial position for only a finite period of time; later, some followers can replace the leader cells that have consumed most of their energy, indicating a dynamic turnover or relay mechanism (Figure 12B; Zhang et al., 2019). Such metabolic regulation is likely to be connected to Notch signaling; future investigations addressing the coupling between signaling, energy consumption and mechanics will be crucial to elucidate the dynamical principles of collective cell migration.

### Mechanosensitivity of Notch Signaling in Cardiovascular Morphogenesis

Evidence of Notch mechanosensitivity in leader-follower cell specification has been observed in a mouse model of retinal angiogenesis, where the Notch1-Dll4 pathway regulates the density of tip cells that give rise to new capillaries from the existing vasculature (Wang et al., 2017). Although lateral inhibition is known to regulate tip/stalk differentiation during branching morphogenesis, this study showed that the tip/stalk differentiation heavily relies on the intercellular tension between cells in the blood vessel (Wang et al., 2017). Similarly to observations in collective epithelial cell migration, tension between cells restricts Notch1-Dll4 signaling and compromises tip cell selection (Wang et al., 2017). Overall, the density of tip cells and new branches was found to negatively correlate with the degree of mechanical stress, suggesting that Notch signaling might be tuned optimally at an intermediate range of intracellular tension that guarantees a proper angiogenic response, but limits the number of new branches (Wang et al., 2017). Interestingly, intercellular tension regulates the Notch-Delta and Notch-Jagged pathways differently in the context of human cardiovascular morphogenesis. Laminar shear stress decreases the expression of Dll4 in human umbilical vein endothelial cells (HUVEC) – as observed in mouse angiogenesis – but also increases the expression of Jagged1, and overall potentiates the signaling between endothelial cells (Driessen et al., 2018).

In the context of cardiovascular morphogenesis, the expression of Notch3, Jagged2 and multiple Notch targets decrease when a higher strain is imposed to vascular smooth muscle cells (VSMCs) (Loerakker et al., 2018). Incorporating the dependence of Notch expression on strain into a computational model shows that the mechanosensitivity of Notch signaling is key in regulating the thickness of the vascular wall. In fact, a switch in cell patterning was observed in a model with an increasing number of VSMCs corresponding to the wall thickness (Loerakker et al., 2018). For a short chain of cells (i.e., thin wall), most cells assumed a Sender state with high Delta. Conversely, thick walls exhibited a chain of cells in a Sender/Receiver state with high Notch and Jagged levels (Loerakker et al., 2018).

The coupling between Notch signaling and mechanical forces is not unidirectional: Notch signaling can, in turn, regulate the function of vascular barriers that separate blood from tissues. For instance, Notch drives the assembly of adherens junctions in a non-canonical mechanism (i.e., not via transcriptional regulation of E-Cadherin levels) (Polacheck et al., 2017). Consistently, reduction of Notch1 due to shear stresses leads to destabilization of adhesion junctions and proliferation of endothelial cells (Mack et al., 2017). Therefore, Notch1 can potentially act as a mechanosensor by regulating the response of endothelial cells based on intercellular stresses, mechanical injuries, and angiogenic signals (Mack et al., 2017). Therefore, while intercellular stresses might fine-tune Notch-Delta/Jagged signaling leading to new vessels, Notch signaling can, in turn, influence the defects in the structure of the vascular barrier by coordinating cell-cell adhesion. Future investigations integrating the interplay between Notch signaling, biomechanical aspects of mechanosensitivity, and the role of cell packing geometry will be valuable in elucidating the emergent dynamics of tissue-level pattern formation in different biological contexts.

## Open Questions and Future Directions

Notch signaling is one of the most ubiquitous transduction pathways in vertebrates. Despite the variety of biological systems and processes, both physiological and pathological, that Notch signaling regulates, its structure and function are incredibly well-conserved.

Notch signaling has drawn incredible attention from the physics and mathematics community because, besides regulating cell-fate at a single cell level, it offers fertile ground to dissect the principles of spatiotemporal pattern formation in a tissue. To the eye of a physicist/mathematician, Notch signaling gives rise to the modes of lateral inhibition and lateral induction similarly to a system of spins that align together or in opposition in a magnet. However, unlike magnetism, these different outcomes of cell states emerge from underlying molecular interactions that are often non-linear and can be separated in time-scale as well. The geometrical intuition about Notch patterning via lateral inhibition and lateral induction provides a key to interpret experimental observations in physiological processes such as embryonic development and angiogenesis (Shaya and Sprinzak, 2011; Bray, 2016; Sjöqvist and Andersson, 2019). For example, lateral inhibition correctly predicts alternate patterning where hair cells (i.e., Senders) are surrounded by supporting cells (i.e., Receivers) and make up about 25% of the total cell fraction, such as in the cases of inner ear development and collective epithelial cell migration (Goodyear and Richardson, 1997; Riahi et al., 2014). Likewise, lateral induction describes well the propagation of similar cell fate observed, for instance, during inner ear development (Petrovic et al., 2014). More investigations, however, will be needed to truly test how well these simple models of biochemical kinetics and feedback loops capture the signaling and patterning dynamics emerging from Notch at a quantitative level.

Moreover, most of the theoretical efforts toward understanding the operating principles of Notch have focused on deterministic models. Cell-to-cell variability, however, can arise due to both stochasticity in the intracellular biochemical signaling (intrinsic noise) and fluctuations of other cellular components and/or in the extracellular environment (extrinsic noise) (Swain et al., 2002). Following a parallel between Notch and other patterning mechanisms driven by nearest neighbor signaling, such as the Ising model for a magnet, we speculate that stochastic fluctuation could play a relevant role in guiding, accelerating and/or disrupting ordered patterns (Rudge and Burrage, 2008).

Additional factors such as cell size and shape, affinity of ligand subtypes, molecular interactions within the same cell, and filopodia modulate the signaling. These mechanisms can be generally seen as details that add further complexity to the simple nearest neighbor’s communication mechanism. For example, it is still not completely understood how trans- and cis-interactions integrate to establish cell fate. Cis-interactions between receptor and ligands of the same cell can typically lead to mutual degradation (Celis and de Bray, 1997; Micchelli et al., 1997; Del Alamo et al., 2011). Recent evidence, however, suggests a role for cis-activation in the Notch pathway for multiple pairs of receptor and ligand subtypes (Nandagopal et al., 2019). Therefore, many context-specific signaling differences and their possible impact on spatiotemporal tissue dynamics deserve finer attention.

Moreover, early experimental findings suggest a role for mechanosensitivity in modulating Notch. The effects of extracellular forces on Notch activation are more quantified at the single molecule level (Meloty-Kapella et al., 2012; Wang and Ha, 2013; Chowdhury et al., 2016); it remains unclear, however, how these effects propagate at the level of multicellular patterning. On the experimental side, novel technologies that allow to probe the spatiotemporal Notch dynamics are starting to provide quantitative insights on the mechanochemical feedbacks between cell-cell signaling and cell mechanics (Riahi et al., 2015; Vishwakarma et al., 2018). On the other hand, integrating aspects of biochemical signaling, mechanical regulation and their interconnections is an important future challenge where theoretical and computational models can assist experimental design and *vice versa*.

Notch signaling has also received attention as a therapeutic target to curb cancer progression (Li et al., 2014; Siebel and Lendahl, 2017). While theoretical modeling of signaling and regulatory dynamics typically adopts modular approaches that treat different signaling modules as independent blocks, Notch seems to be implicated in several hallmarks of cancer progression, including drug-resistance, leaky/chaotic angiogenesis and enhanced invasion and metastasis (Li et al., 2014; Siebel and Lendahl, 2017). Jag1 is highly expressed in circulating tumor cell clusters with higher metastatic potential (Jolly et al., 2017) and by cancer cells that resist to drugs (Boareto et al., 2016; Yang et al., 2019). Generally speaking, cells that highly express Jagged seem to be associated with a more plastic and undifferentiated state such as hybrid epithelial/mesenchymal and/or a stem-like phenotype (Wang et al., 2009; Sikandar et al., 2010; Zhu et al., 2011; Yamamoto et al., 2013; Bocci et al., 2019a). Therefore, quantifying the role of interconnections between Notch and other hallmarks of cancer invasion will be a crucial challenge at the crossing point between theoretical modeling, biology and data science.

Another exciting direction concerns the widespread development of single cell sequencing techniques. Recently, Notch signaling has been studied at the single cell resolution in Zebrafish development, hematopoiesis and cancer stem cells (Mark et al., 2019; Tikhonova et al., 2019; Annika et al., 2020). For instance, it has been observed that downregulation of Notch ligands Dll1 and Dll4 in the bone marrow correlates with premature activation of a myeloid transcriptional program in hemopoietic stem cells (Tikhonova et al., 2019). While these studies provide detailed information on the transcriptional dynamics of Notch, they still lack the spatial resolution necessary to elucidate the underlying patterning mechanisms. Certainly, our understanding of Notch signaling will benefit from future developments in the field of single cell sequencing to account for spatial patterning.

Overall, insights from experimental and theoretical models continue to unravel the operating principles of Notch signaling, a master regulator of spatiotemporal cell patterning in development and tumor progression.

## Author Contributions

All authors discussed the content of the manuscript, agreed to be accountable for the content of the work, and discussed and edited the manuscript. FB wrote the manuscript. JO and MJ led the research.

## Conflict of Interest

The authors declare that the research was conducted in the absence of any commercial or financial relationships that could be construed as a potential conflict of interest.
